# Estimation of In Vitro True Digestibility and Fiber Degradation from Feedstuff Fiber Composition When Incubated in Equine Fecal Inoculum

**DOI:** 10.3390/ani13233699

**Published:** 2023-11-29

**Authors:** Ryon W. Springer, Nichole M. Cherry, Randel H. Raub, Kimberly B. Wellmann, Trinette N. Jones

**Affiliations:** 1 Department of Animal Science, Tarleton State University, Stephenville, TX 76402, USA; kwellmann@tarleton.edu (K.B.W.); tnjones@tarleton.edu (T.N.J.); 2 Texas A&M AgriLife Research, Stephenville, TX 76401, USA; nichole.cherry@ag.tamu.edu; 3 Kent Nutrition Group, Muscatine, IA 52761, USA; randel.raub@kentww.com

**Keywords:** neutral detergent fiber, acid detergent fiber, digestibility modeling, Akaike’s information criterion, goodness-of-fit

## Abstract

**Simple Summary:**

Feedstuff fiber composition is known to affect digestibility of feeds within the horse. Neutral detergent fiber (NDF) is a chemical measure of plant cell wall material to estimate the amount of feed that will reach the hindgut of the gastrointestinal tract. Acid detergent fiber (ADF) estimates the amount of NDF that is largely unavailable for microbial fermentation in the hindgut of the horse due to the structure of the cell wall. Increases in both NDF and ADF are known to decrease total digestibility in the horse and other species of animals. This study’s objective was to determine if feedstuff NDF and ADF could predict simulated digestion in the horse using fecal microbes. Prediction models showed that the interaction of NDF and ADF best predicted total digestion of a feedstuff. However, NDF composition was a poor predictor of fiber digestion by hindgut microbes. Feedstuff ADF strongly predicted fiber degradation in the hindgut as it estimated the amount of cell wall that is resistant to microbial fermentation. The fiber composition of feedstuffs can be used to estimate total digestion and fiber degradation under simulated conditions mimicking the hindgut of the horse.

**Abstract:**

Neutral detergent fiber (NDF) and acid detergent fiber (ADF) composition have been shown to predict in vitro true digestibility (IVTD), in vitro NDF digestibility (IVNDFD), and in vitro ADF digestibility (IVADFD) in ruminants. This study’s objective was to estimate in vitro digestibility measures within the Daisy^II^ incubator using equine fecal inoculum from feedstuff NDF and ADF composition. Analyzed feedstuffs included alfalfa hay (*Medicago sativa*), Coastal Bermudagrass hay, soybean meal, rice bran, hempseed meal, and Bluebonnet® Equilene® Pellets. Data were analyzed using Akaike’s information criterion (AIC) within the R Statistical Program©. The highest ranked model for IVTD was the interaction of NDF and ADF: 10003.32 – 0.2904 × NDF − 0.4220 × ADF − 0.0010 × NDF × ADF (Adjusted R^2^ = 0.959 and AICc = 474.97). Sample IVNDFD was moderately predicted by ADF: 855.15 – 1.5183 × ADF (Adjusted R^2^ = 0.749 and AICc = 560.82). Feedstuff ADF produced the highest ranked model for IVNDFD: 881.91 – 1.5952 × ADF (Adj. R^2^ = 0.835 and AICc = 541.33). These results indicate the effectiveness of using feedstuff NDF and ADF composition to predict IVTD, IVNDFD, and IVADFD within equine fecal inoculum. The findings of this study provide better understanding of feedstuff digestibility using equine fecal inoculum, but more research is warranted for validation of the models and the potential impact in vivo.

## 1. Introduction

Nutrition trials typically measure in vivo apparent digestibility of nutrients within feedstuffs because endogenous losses that occur within the gastrointestinal tract cannot always be measured. However, true digestibility accounts for endogenous losses and provides a more controlled measurement of digestibility [1]. Previous research has established estimates of in vivo true digestibility for various nutrients (i.e., non-structural carbohydrates, crude protein, and neutral detergent fiber) within equine feedstuffs [2]. The application of in vitro methods within the Daisy^II^ incubator (ANKOM Technology Corporation, Fairport, NY, USA) has been validated to estimate feedstuff digestibility in hindgut fermenters such as horses [3,4], donkeys [5], and rabbits [6] with a variety of applications for digestibility research [7]. Previous studies support using fresh feces as a microbial inoculum source to simulate hindgut digestion in the horse compared to in vivo trials [3,4,8]. Although the microbial population of the hindgut changes greatly from the cecum to the dorsal colon [9], feces as a microbial source does not differ in fermentation capacity in vitro as compared to inoculum obtained from the upper regions of the hindgut such as the cecum and ventral colon [10,11]. Nutrient composition variables (i.e., crude protein, neutral detergent fiber, and acid detergent fiber) have been used to estimate in vivo dry matter digestibility of hays in horses [12]. Fonnesbeck [13] concluded that neutral detergent fiber (NDF) is indicative of gut fill due to its inclusive chemical composition of hemicellulose, pectin, cellulose, and lignin while Keys et al. [14] indicated that acid detergent fiber (ADF) and its components (cellulose and lignin) were more indicative of fiber digestion in the hindgut of the gastrointestinal tract. Hemicellulose and ADF are used within a multiple regression model to estimate digestible energy in horses. Hemicellulose is available for microbial fermentation while lignification within ADF limits digestibility in the hindgut [15]. The fiber composition of hemicellulose and ADF had a negative impact on feedstuff digestible energy content due to lower digestibility [16]. Although there are studies validating the use of fiber composition to estimate in vivo digestibility, few have attempted modeling in vitro true digestibility (IVTD), in vitro neutral detergent fiber digestibility (IVNDFD), or in vitro acid detergent fiber digestibility (IVADFD) in horses using NDF and ADF as predictor variables. The current status quo within animal nutrition is to use goodness-of-fit to explain data variation and produce single-best models. However, several authors in the field of wildlife management and ecology have demonstrated the benefits of using Akaike’s information criterion (AIC) as a standard for model selection and determining and predictor variable power [17,18,19,20]. However, there is still merit in using a combination of goodness-of-fit to evaluate variable explanatory power and AIC to compare the predictor power of the model [21]. Thus, the primary objective of this study was to determine if feedstuff IVTD, IVNDFD, and IVADFD could be determined by using feedstuff NDF and ADF composition when incubated in equine feces as a microbial inoculum within the Daisy^II^ incubator (ANKOM Technology Corporation, Fairport, NY, USA). A secondary objective was to demonstrate the use of AIC model selection and compare the results to that of goodness-of-fit selection.

## 2. Materials and Methods

### 2.1. Animals and Diets

All methods described were approved by the Tarleton State University Institutional Animal Care and Use Committee (Protocol #03-008-2022). An in vitro split-split-plot design was conducted using two Daisy^II^ (ANKOM Technology Corporation, Fairport, NY, USA) incubators (*n* = 2), four mature Quarter Horse geldings as microbial inoculum sources (*n* = 4), and six different feedstuffs (*n* = 6). This resulted in each horse being represented by one jar in each incubator, with all feed samples represented in each incubator jar. The horses were selected from the Tarleton State University’s herd located in Stephenville, TX. Horses were 8.5 ± 3.9 years of age, weighed 558.4 ± 3.9 kg, and had a body condition score [22] of 6.0 ± 0.5 on the day of fecal collection. Horses underwent a 21-day acclimation period prior to fecal collection to standardize both diet and environment between horses [3]. Horses were housed in 10 m × 20 m dry lot runs at the Tarleton State University Equine Center (Stephenville, TX, USA). Basal diets consisted of 1.8% body weight as-fed of Coastal Bermudagrass hay (*Cynodon dactylon*) and 0.2% body weight as-fed of Bluebonnet® Equilene® Pellets (Bluebonnet® Feeds, Ardmore, OK, USA). The basal diet consisted of 683.9 g/kg dry matter (DM) NDF and 332.0g/kg DM ADF. Meals were divided into two equal feedings at 0800 h and 1600 h each day. Water and salt blocks were provided ad libitum.

### 2.2. Feedstuff Sample Preparation

Approximately 400 g of each feedstuff was collected: Alfalfa hay (*Medicoago sativa*), Coastal Bermudagrass hay (*Cynodon dactylon*), hempseed meal pellets (*Cannabis sativa* L.), soybean meal pellets (*Glycine max* L.), rice bran pellets (*Oryza sativa* L.), and Bluebonnet® Equilene® Pellets (EQU; Bluebonnet® Feeds, Ardmore, OK, USA). Samples were weighed then dried overnight in a forced-air drying oven at 55 °C. Sample weights were collected again to determine dry matter (g/kg), then passed through a 2 mm screen of a Wiley Mill (Arthur H. Thomas Co., Philadelphia, PA, USA). ANKOM F57 filter bags (ANKOM Technology Corporation, Fairport, NY, USA) were labeled, rinsed in acetone, dried, and weighed. Feedstuff samples were weighed out to 0.5 g then heat-sealed in the ANKOM F57 filter bags (Fairport, NY, USA). Each Daisy^II^ incubator jar (ANKOM Technology Corporation, Fairport, NY, USA) contained two filter bags of each experimental feedstuff, one EQU and one blank bag to use as a correction factor. A duplicate set of filter bags with samples were set aside for pre-incubation NDF and ADF analysis using the ANKOM^200^ Fiber Analyzer (ANKOM Technology Corporation, Fairport, NY, USA).

### 2.3. Fecal Sample Collection

Fresh feces were collected on day 22 of this study at 0900 h via rectal grab or immediately after defecation by the same researcher. The fecal collection occurred 1 h after the morning feeding. A minimum sample of 400 g of feces was collected from each horse to ensure an adequate amount for inoculum preparation. All samples were immediately placed into resealable plastic bags and air was forced out to decrease air exposure to the microbes. For transportation to the laboratory, fecal collection bags were placed in thermoses that were warmed to 39 °C using water [3,4]. Fecal samples were transported to the laboratory and used as inoculum sources within 1 h post-collection.

### 2.4. Fecal Inoculum Preparation

Two buffers were pre-warmed to 39 °C prior to fecal preparation. Buffer A was measured out to 1330 mL while Buffer B was measured out to ~266 mL [23]. Both buffers were mixed, and the pH was adjusted using either Buffer B or 2NHCl to adjust the pH to approximately 7.0. Jars were placed back into the preheated incubator until fecal inoculum preparation. Individual fecal samples from each horse were measured to 200 g on a scale for each incubator and individually placed into a blender (Hamilton Beach®, Glen Allen, VA, USA) and mixed with 400 mL of mixed buffer solution. Once blended, fecal solutions were strained through cheesecloth into a plastic container. Filter bags (*n* = 12/jar) were placed into an incubator jar and strained fecal solution was poured into the jar. Each jar was purged with CO_2_ for 30 s before being placed in the Daisy^II^ incubator (ANKOM Technology Corporation, Fairport, NY, USA). This process was repeated until all jars (*n* = 8) were placed in incubation for 48 hours [3,4]. 

### 2.5. Chemical Analysis

Split feedstuff samples were used to determine pre-digestion NDF and ADF concentrations. The NDF and ADF composition of each feedstuff were measured with two ANKOM^200^ Fiber Analyzers (ANKOM Technology Corporation, Fairport, NY, USA) using methods described by ANKOM Technology [24]. After the 48-hour incubation period, filter bags were washed using cold tap water until water was clear, then placed in the ANKOM^200^ Fiber Analyzers using the methods described for pre-incubation NDF and ADF concentrations.

### 2.6. Calculations

The sample weights obtained from pre- and post-incubation were used in the following equations to calculate in vitro digestibility measurements modified from ANKOM Technology [23] and Tassone et al. [5].

In vitro true digestibility:

(IVTD, g/kg DM) = 1000 × (DM_0h_ − (NDF_residue_ − Blank_correction_))/DM_0h_ (1)

In vitro NDF digestibility:

(IVNDFD, g/kg NDF) = 1000 × (NDF_0h_ − (NDF_residue_ − Blank_correction)_)/NDF_0h_ (2)

In vitro ADF digestibility:

(IVADFD, g/kg ADF) = 1000 × (ADF_0h_ − ADF_residue_ − Blank_correction_)/ADF_0h_ (3)

where the terms are described as follows:

DM_0h_ = Initial sample weight (g DM—dry matter);
NDF_0h_ = Split sample weight after neutral detergent treatment (g NDF);
Blank_correction_ = Final oven-dried weight (g DM)−original blank bag weight (g DM);
NDF_residue_ = Final bag weight after incubation and neutral detergent treatment (g NDF);
ADF_0h_ = Split sample weight after neutral and acid detergent treatments (g ADF);
ADF_residue_ = Final bag weight after incubation and detergent treatments (g ADF).

### 2.7. Statistical Analysis

#### 2.7.1. Data Management and Model Development

Data used for model development were obtained from Springer et al. [25] when investigating the in vitro digestibility of hempseed meal using equine feces as microbial inoculum. The ranges for fiber composition and in vitro digestibility of each feedstuff are presented in Table 1. Statistical analysis and data management were performed within the R Statistical Program© (v4.2.2; R Core Team, Vienna, Austria). Feed samples that were placed in duplicate within each incubator jar and fiber analyzer were averaged for statistical analysis resulting in *n* = 8 for each sample and 48 total feed samples. Data were normalized to 1.5× the interquartile range for each digestibility variable. A two-way analysis of variance (ANOVA) was used to determine the effects of horse and Daisy^II^ incubator (ANKOM Technology Corporation, Fairport, NY, USA) on digestibility coefficients. Linear regression models were developed using NDF, ADF, and NDF × ADF as predictor variables for estimation of in vitro digestibility. The mixed model of NDF × ADF is considered the “global model”; thus, although maybe not biologically relevant, it is applied to all digestibility measures for modeling and statistical purposes [26]. A two-way ANOVA was used to determine differences in model slopes between feedstuffs. Differences between slope coefficients were determined using least-squares means. The adjusted R^2^ (Adj. R^2^) and residual standard error (RSE) for each model were used to determine explanatory power of the predictor variables within the dataset. Models were considered to have moderate and high explanatory power when Adj. R^2^ > 0.7 and Adj. R^2^ > 0.85, respectively. Models within a given response variable with a smaller RSE were considered to have greater explanatory power for the given data.

#### 2.7.2. Model Selection Using Akaike’s Information Criterion (AIC)

Models were developed using Akaike’s information criterion (AIC) as described by Burnham and Anderson [19] using the AICcmodavg package [26] to determine predictor variable power and rank the resulting models within a response variable. The AIC method uses each model’s maximum likelihood estimation (Log-Likelihood: LL) as a measure of data fit. A model’s LL is inversely related to its AIC meaning that a higher LL results in a lower AIC. Thus, a lower AIC indicates the model fits the data better when compared to other models for the same dataset. The AIC method also rewards simpler models because the use of a greater number of parameters is more likely to overfit the given dataset. The calculation for AIC is as follows:

AIC = 2*k* − 2ln(LL) (4)

where the terms are described as follows:

ln = natural log;
LL = log-likelihood of the model;
*k* = (number of parameters) + 2.

Ranking models by AIC allows for model development using small sample sizes (*n*/*k* < 40) that prevent further model validation. Model AIC can be corrected for large sample sizes using a correction term resulting in a conservative AIC score (AICc). The use of AICc allows for conservative application of the model to larger datasets. The calculation of AICc is as follows:

AICc = AIC + 2*k*(*k* + 1)/(*n* − *k* + 1) (5)

where the terms are described as follows:

AIC: Akaike’s information criterion Score;
*k* = (number of parameters) + 2;
*n* = sample size.

Delta AICc (∆AICc) is a metric to determine differences in evidence for the predictive power of each model for the dataset. The ∆AICc for each model is calculated by subtracting the AICc of the highest-ranked model from the model in question. Typical thresholds for determining model strength are as follows:

- ∆AICc < 2: substantial evidence for selected model;
- ∆AICc < 7: moderate support for selected model;
- ∆AICc < 10: low support for selected model;
- ∆AICc > 10: model is very unlikely to predict the given data.

Another metric is AICc weights (AICcWt) and cumulative weights (Cum.Wt). The AICcWt is the ratio of ∆AICc of the individual model to the whole set of candidate models. The AICcWt is indicative of the probability that the individual model is best among all candidate models. The Cum.Wt is the sum of individual model and the higher-ranking models’ AICcWt to provide the percent of the predictive power for all the top-ranking models. Significance for variable slope was set at *p* ≤ 0.05 with trends considered at 0.05 < *p* ≤ 0.10. Models were ranked using model ∆AICc, AICcWt, and biological relevance while using goodness-of-fit slope to determine the significance of the predictor variables within the individual response variables [17,21,26]. 

## 3. Results

There was no effect of Daisy^II^ (ANKOM Technology Corporation, Fairport, NY, USA) incubator (*p* ≥ 0.898) or horse (*p* ≥ 0.893) on feedstuff in vitro digestibility. Slopes did not differ between feedstuffs for IVTD (*p* = 0.536), IVNDFD (*p* = 0.855), or IVADFD (*p* = 0.396). The coefficients of the linear and multiple linear models predicting IVTD, IVNDFD, and IVADFD using feedstuff NDF and ADF composition are shown in Table 2.

**Figure 1:**
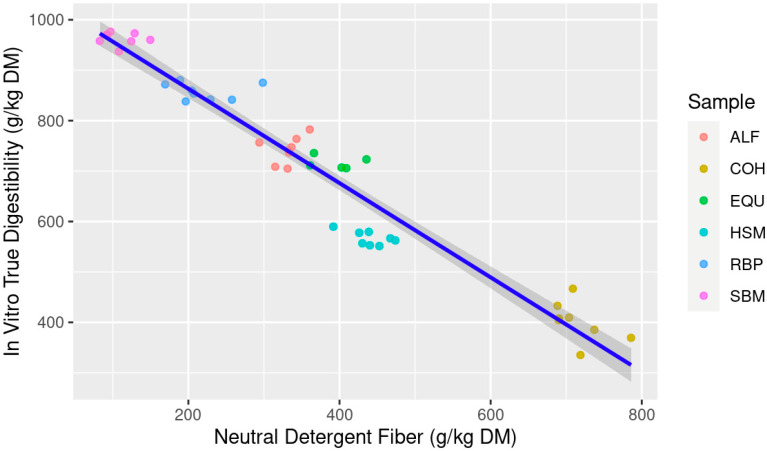
Estimation of in vitro true digestibility (IVTD; g/kg dry matter (DM)) from feedstuff neutral detergent fiber (NDF; g/kg DM) composition using equine feces as incubation inoculum; Abbreviations: ALF: Alfalfa hay; COH: Coastal bermudagrass hay; EQU: Bluebonnet® Equilene® Pellets (Bluebonnet® Feeds, Ardmore, OK, USA); HSM: Hempseed meal pellets; RBP: Rice bran pellets; SBM: Soybean meal.

**Figure 2:**
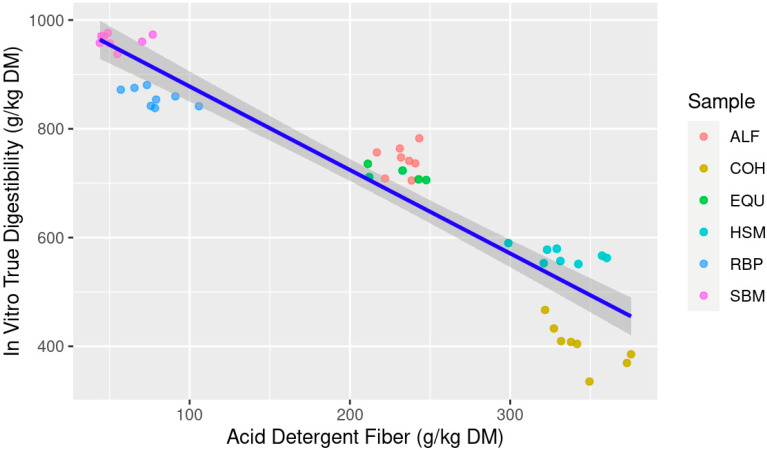
Estimation of in vitro true digestibility (IVTD; g/kg dry matter (DM)) from feedstuff acid detergent fiber (ADF; g/kg DM) composition using equine feces as incubation inoculum; Abbreviations: ALF: Alfalfa hay; COH: Coastal bermudagrass hay; EQU: Bluebonnet® Equilene® Pellets (Bluebonnet® Feeds, Ardmore, OK, USA); HSM: Hempseed meal pellets; RBP: Rice bran pellets; SBM: Soybean meal.

When ranking the models predicting IVTD, the multiple linear regression best predicts future data with the lowest AICc and most negative LL and explains current data with the highest adjusted R^2^ and lowest RSE compared to the simple linear regressions. The ΔAICc for both NDF and ADF as individual predictor variables are beyond the threshold of 10; thus, they are classified as very unlikely to predict future data which is also supported by the lower LL for both simple regression models. The AICcWt and Cum.Wt for the mixed model are 1.0, indicating 100% of the evidence is in favor of the mixed model compared to the simple models. However, when comparing the simple linear regressions, NDF was the best explanatory variable for IVTD versus ADF (Table 3). When both linear models are visualized, NDF content was much more evenly distributed along the *x*-axis (Figure 1) compared to ADF (Figure 2). It is important to note that all variables within the models presented for IVTD were significant (*p* < 0.05) indicating contribution of each variable (NDF and ADF) to the model itself, thus the prediction and explanatory power of the model indicate that the multiple regression model of NDF × ADF is best suited.

**Figure 3:**
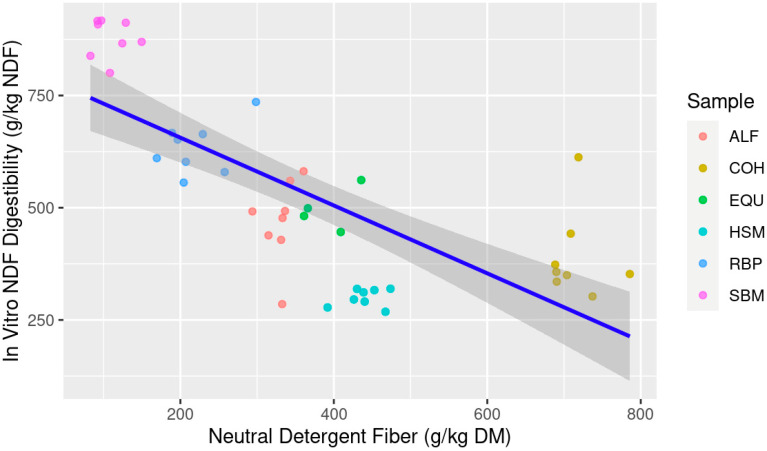
Estimation of in vitro neutral detergent fiber digestibility (IVNDFD; g/kg NDF) from feedstuff neutral detergent fiber (NDF; g/kg dry matter (DM)) composition; Abbreviations: ALF: Alfalfa hay; COH: Coastal bermudagrass hay; EQU: Bluebonnet® Equilene® Pellets (Bluebonnet® Feeds, Ardmore, OK, USA); HSM: Hempseed meal pellets; RBP: Rice bran pellets; SBM: Soybean meal.

**Figure 4:**
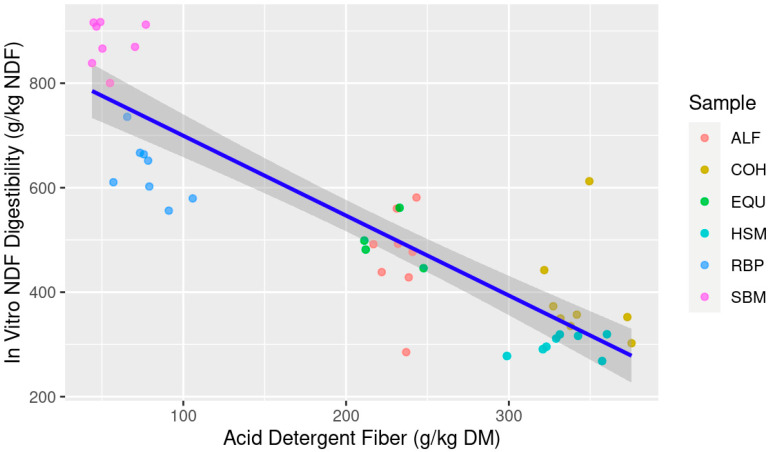
Estimation of in vitro neutral detergent fiber digestibility (IVNDFD; g/kg NDF) from feedstuff acid detergent fiber (ADF; g/kg dry matter (DM)) composition; Abbreviations: ALF: Alfalfa hay; COH: Coastal bermudagrass hay; EQU: Bluebonnet® Equilene® Pellets (Bluebonnet® Feeds, Ardmore, OK, USA); HSM: Hempseed meal pellets; RBP: Rice bran pellets; SBM: Soybean meal.

The linear and multiple linear regression models used to estimate IVNDFD are presented in Table 4 while simple linear models for NDF and ADF are shown in Figure 3 and Figure 4, respectively. As seen with IVTD, IVNDFD was best predicted with NDF × ADF using both goodness-of-fit and AIC metrics. However, the simple linear model showed that ADF was the best single predictor as compared to NDF. When models were weighted, the NDF × ADF model had an AICcWt of 0.89 compared to ADF with 0.11 resulting in the Cum.Wt being 1.0 between the two models. The mixed model also had the greatest Adj. R^2^ and lowest RSE compared to the simple models. However, NDF as a single variable was low in both predictive power (AICcWt = 0.0), falling well beyond the threshold of ΔAICc = 10, and only explains approximately 50% of the given data (adj. R^2^ = 0.524) (Table 4; Figure 3). However, even though the mixed model shows greater predictive power, the predictor variable of NDF in the model IVNDFD~NDF × ADF was not significant (*p* > 0.05), thus the best model would default to IVNDFD~ADF due to its AICcWt, Adj. R^2^ value, and RSE.

**Figure 5:**
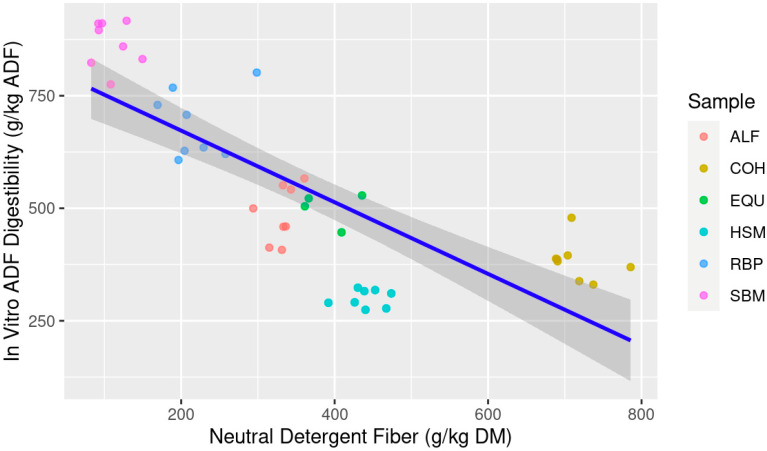
Estimation of in vitro acid detergent fiber digestibility (IVADFD; g/kg ADF) from feedstuff neutral detergent fiber (NDF; g/kg dry matter (DM)) composition; Abbreviations: ALF: Alfalfa hay; COH: Coastal bermudagrass hay; EQU: Bluebonnet® Equilene® Pellets (Bluebonnet® Feeds, Ardmore, OK, USA); HSM: Hempseed meal pellets; RBP: Rice bran pellets; SBM: Soybean meal.

**Figure 6:**
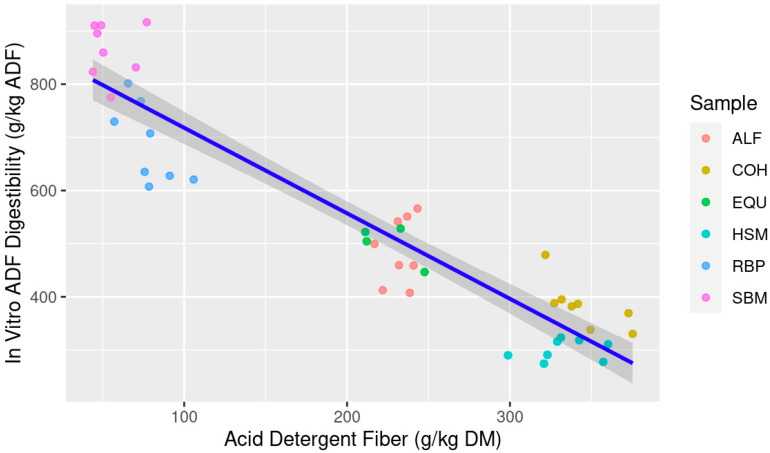
Estimation of in vitro acid detergent fiber digestibility (IVADFD; g/kg ADF) from feedstuff acid detergent fiber (ADF; g/kg dry matter (DM)) composition; Abbreviations: ALF: Alfalfa hay; COH: Coastal bermudagrass hay; EQU: Bluebonnet® Equilene® Pellets (Bluebonnet® Feeds, Ardmore, OK, USA); HSM: Hempseed meal pellets; RBP: Rice bran pellets; SBM: Soybean meal.

The resulting models of IVADFD found in Table 1 are ranked by predictor power in Table 4 with simple linear regressions from NDF and ADF composition shown in Figure 5 and Figure 6, respectively. Within Table 5, the multiple linear regression demonstrated the highest predictor power of the three models (AICcWt = 0.8), resulting from the greatest LL and having the highest explanatory power (Adj. R^2^ = 0.854). However, the simple linear model using ADF as the predictor variable carries weight due to its similar predictor (AICcWt = 0.2) and explanatory power (Adj. R^2^ = 0.834). The ΔAICc of the simple ADF model was also below the threshold of 4, thus indicating moderate support for the model. Between the mixed model and simple ADF model, the Cum.Wt was 1.0, indicating the data support resides within the two models collectively. Neutral detergent fiber as a predictor variable for IVADFD is not greatly powerful in either prediction (AICcWt = 0.0; ΔAICc > 10) or explanation of the given data (Adj. R^2^ = 0.586). Within the multiple linear regression model for IVADFD, NDF as a predictor variable was not significant (*p* > 0.05); thus, with its similar predictor and explanatory power, the best option was using ADF as the predictor variable within a simple regression.

## 4. Discussion

The primary objective of this study was to determine if feedstuff fiber composition (NDF and ADF) could be used to develop models to predict in vitro true digestibility (IVTD) and fiber degradation (IVNDFD and IVADFD) when incubated using equine fecal inoculum. Neutral detergent fiber (NDF) chemically distinguishes between neutral detergent solubles (NDS) and cell wall constituents within a feedstuff. Thus, NDF and NDS are inversely related within the plant cell. In the horse, as feedstuff NDF content increases, total digestibility of the feedstuff decreases due to decreased NDS [27]. This is due to the differences in digestibility between NDF and NDS. Neutral detergent solubles include soluble protein, soluble carbohydrates (non-structural carbohydrates), and fatty acids that are readily digested in the foregut of the horse [2,13,15,27,28]. Pagan [2] reported estimated true digestibility for NDF, soluble carbohydrates, and crude protein in the horse at 45.0%, 97.4%, and 86.1%, respectively. Van Soest [15] found that the NDS portion of feedstuffs were highly digestible due to the lack of lignification that is only found within NDF. These findings support the notion that total digestibility of a feedstuff decreases as NDF content increases due to the decreased digestibility of the NDF portion. However, a previous study using rumen fluid found that NDF was not as strong of a predictor variable for IVTD in tropical plants (R^2^ = −0.69) as ADF (R^2^ = −0.83) or lignin (R^2^ = −0.80) when performing a simple linear regression. However, when a multiple regression was performed, NDF explained 48% of the variance while ADF only explained 2% indicating that NDF may be a good predictor across feedstuffs, but ADF allows for greater explanation within a specific feedstuff due to differences in hemicellulose content and thus fiber degradation [29]. Other studies report an explanatory power of NDF for IVTD to be strong as well, with R^2^ ranging from −0.77 to −0.87 when incubated in rumen fluid [30,31]. However, Henderson and Robinson [32] reported that lignin was the best predictor of IVTD (r = −0.63 to −0.91) compared to cellulose or ADF while Claessens et al. [30] found that ADL was better at explaining IVTD (R^2^ = −0.96) than ADF (R^2^ = −0.88) and NDF (R^2^ = −0.87). However, this study indicated that NDF was the best predictor compared to ADF in both explanatory and predictor power. When comparing the current models to those developed by Arroyo-Aguila and Coward-Lord [29], the slope for NDF as a predictor variable was greater in the other study (−1.34) compared to ours (−0.9325) while the slopes for ADF as a predictor variable were similar (−1.52 vs. −1.5282). While both studies indicate a negative impact of NDF on digestibility, inferred by a negative variable slope, Arroyo-Aguila and Coward-Lord [29] indicates greater detriment of NDF on IVTD than our study. This may be due to differences in microbial inoculum as well as fiber composition of the feedstuffs used within the experiments. Overall, NDF may be a better predictor for IVTD than ADF as NDF quantifies both cell wall contents and cell contents while ADF was originally only intended as a preparation stage for quantification of cellulose, lignin, and silica, all of which are determinants in cell wall degradability [28]. The interaction of NDF × ADF can be interpreted as the proportion of NDF unavailable for digestion, or contrarily, the amount of hemicellulose that is available to the microbes [14]. Neutral detergent fiber (NDF) is best used to indicate the location of digestion of a feedstuff [13]. As NDF increases, the primary location of feedstuff digestion shifts toward the hindgut of the animal as NDF is highly indigestible in the foregut. However, ADF indicates the digestibility of the NDF portion within the hindgut as it determines the amount of fiber available for microbial fermentation [14,15]. Overall, the interaction of NDF × ADF best explains the gastrointestinal physiology and feed digestibility within the horse.

Regarding fiber degradation, previous research in horses has found that neutral detergent fiber digestibility (NDFD) variations between feedstuffs is due to the composition of the substrates that compose NDF [27]. Neutral detergent fiber is composed of hemicellulose, cellulose, and lignin while ADF comprises only cellulose and lignin [15]. Due to the large inclusivity of NDF, its composition is widely variable, and thus, may not be as robust to use as a predictor variable to determine fiber degradation [13]. This study found that ADF was a better predictor variable for both IVNDFD (R^2^ = 0.749) and IVADFD (R^2^ = 0.835) compared to NDF. This is likely due to NDF being an indicator of gastrointestinal fill while ADF is indicative of fiber quality [13]. Hemicellulose, calculated as NDF minus ADF, has greater digestibility than cellulose, calculated as ADF minus ADL, in non-ruminants (pigs and rats), indicating that fiber quality rather than quantity may determine the degree of fiber degradation [14]. Neutral detergent fiber digestibility is negatively correlated with NDF content of hays in horses [33] but has been shown to be an indicator of fill in ruminants with little impact on NDFD due to its content variability [Getachew et al., 2004]. Acid detergent lignin (ADL), a structural non-carbohydrate, is deposited in the cell wall after hemicellulose and cellulose to increase structural integrity of the cell wall. The incorporation of ADL into the cell wall acts as a physical barrier to microbial fibrolytic enzymes, thus reducing fiber digestibility [34]. Claessens et al. [30] reported that ADL was the best explanatory variable for IVNDFD in rumen fluid. The authors found that ADL had the highest explanatory power (R^2^ = −0.82) compared to ADF (R^2^ = −0.67) and NDF (R^2^ = −0.65). Acid detergent lignin is highly indigestible; thus, it can be used a predictor variable for fiber degradation [29]. Although digestibility of ADL is relatively low (~200 to 300 g/kg ADL), recent research in horses has shown that small decreases in forage ADL result in increases in apparent NDF and ADF digestibility [35]. In lambs, NDFD has been determined in vivo by ADL content with moderate explanatory power (r = −0.73) [36]. However, when ADL was used as a fraction of NDF as a predictor variable (ADL/NDF), the explanatory power was increased (ADL: R^2^ = −0.50; ADL/NDF: R^2^ = −0.55) [37]. However, unlike other studies, ADL was not analyzed, only ADF. Still, ADF was a powerful predictor variable for both IVNDFD and IVADFD with the current multiple linear regressions using both ADF and NDF resulting in insignificance of the NDF as a variable. Thus, ADF may be useful in predicting fiber degradation in horses and alleviating the need for further chemical analysis.

Previous studies have shown that fecal donor diets impact the fermentation capacity of the microbial inoculum [3,38]; specifically, that high-fiber diets reduce the microbial variability in the hindgut of the horse [39,40]. However, increasing soluble carbohydrates in the diet increases NDF digestibility in vivo in both ruminants and horses, likely due to the increased microbial diversity within the gastrointestinal tract [9,41]. The concentration of the diet provided to the fecal donor horses was 683.9 g/kg DM NDF and 332.0 g/kg DM ADF. Because of the fiber-dense diet consumed by the horses in this study, the variations observed in the fiber degradation models may be attributed to the microbial population of the inoculum, thus donor diets containing higher soluble carbohydrates or lower NDF and ADF may produce different statistical models. Furthermore, in vivo and in vitro digestibility trials do not produce identical results [3,6,8]; thus, caution is warranted when translating the present in vitro models to an in vivo setting. Future investigation is warranted to determine if the models produced in this study are applicable using microbial inoculum from horses consuming diets of differing nutrient composition while also determining their relevance in vivo.

The use of AIC model selection has become highly prevalent in ecological journals such as “*Ecology*”, “*Journal of Wildlife Management*”, and “*Journal of Applied Ecology*” [42], but is not common in areas of animal science, more specifically animal nutrition. The typical method of model selection within animal nutrition has been the use of goodness-of-fit with many parameters that may over-fit the data. Although this paper used goodness-of-fit as a standard parameter, effort was made to distinguish between explanatory power (Adj. R^2^ and RSE) and that of predictor power (AICc, ∆AICc, and AICcWt) and potential differences that occurred between the two methods [21]. Burnham and Anderson [18] demonstrated the benefits of using AIC as a one-dimensional method of model selection with a standard set of criteria and the need to optimize the use of biologically relevant predictor variables. Furthermore, the typical use of multiple regressions with many parameters should be considered the global model (the “inclusive” model) and not necessarily the optimal model, while more simplistic models may demonstrate greater biological relevance. Many multiple regression equations tend to include parameters that are not necessarily pertinent to the physiological aspects of the study, but rather increase the explanatory power of the model [20,21]. This is seen with using ADF as a predictor variable in both IVNDFD and IVADFD estimation. The global model of NDF × ADF produced some relative explanatory power, but NDF itself contributed little to the models. There is little biological relevance of NDF within both models for predicting fiber degradation as NDF is a measurement of the fiber itself, while ADF is measure of fiber that is less digestible by the hindgut microbes [13,14]. Furthermore, the simple regression models using ADF provided adequate predictor power with their AICc and AICcWt outputs as well as explanatory power via Adj. R^2^ and RSE. Overall, using AIC methods of model selection alongside goodness-of-fit may further advance the statistical power of modeling within the field of animal nutrition. 

## 5. Conclusions

The horse has a unique digestion system compared to other nonruminants; thus, it is not conducive to use digestibility models in other species to directly estimate feedstuff digestion in horses. In vivo trials can be difficult and expensive to perform, thus in vitro methodology can be used in its place for approximation of digestibility. Digestibility modeling can also provide efficient methods for estimating nutritional value of feedstuffs, specifically energy concentrations. This study found that IVTD could be best estimated with the mixed model of ADF and NDF, but the single factor of NDF is of great significance as well. Regarding fiber degradation, ADF was the single best predictor variable for both IVADFD and IVNDFD as it is an estimate of fiber quality. When NDF and ADF were used in multiple linear regression models to estimate fiber digestibility, NDF was not significant resulting in ADF as a better model. Feedstuff NDF and ADF composition can be used to predict IVTD and fiber degradation when using equine feces as a microbial inoculum. Although using AIC as a method of model selection is not a novel concept, it is not commonly used in the field of animal nutrition. The statistical methodology described in this paper may provide a simplistic method of nutritional modeling to enhance the statistical power and biological relevance of future nutrition models. Modeling using AIC may also be beneficial when using small datasets (*n*/*k* < 40) without resources for model validation.

## Figures and Tables

**Table 1:** Range (minimum and maximum) of feedstuff fiber composition and in vitro digestibility measures used for model development to in vitro true digestibility and fiber degradation from fiber composition.

Feedstuff	NDF	ADF	IVTD	IVNDFD	IVADFD
ALF	293.9–360.6	216.8–243.3	704.9–782.5	285.2–581.2	407.5–566.2
COH	688.5–785.8	321.8–375.4	335.3–466.8	302.4–612.4	330.4–478.9
EQU	361.2–435.7	211.2–269.6	705.8–972.6	446.0–959.9	446.6–983.3
HSM	391.9–473.9	298.8–360.3	551.3–589.8	268.2–319.5	274.4–323.5
RBP	169.3–298.6	57.1–105.8	838.1–880.5	556.1–735.7	607.3–801.5
SBM	82.8–149.6	44.0–77.0	937.4–976.5	800.3–917.1	775.2–916.6

Abbreviations: NDF: neutral detergent fiber (g/kg dry matter (DM)); ADF: acid detergent fiber (g/kg DM); IVTD: in vitro true digestibility (g/kg DM); IVNDFD: in vitro neutral detergent fiber digestibility (g/kg NDF); IVADFD: in vitro acid detergent fiber digestibility (g/kg ADF); ALF: Alfalfa hay; COH: Coastal Bermudagrass hay; EQU: Bluebonnet® Equilene® Pellets (Bluebonnet® Feeds, Ardmore, OK, USA); HSM: Hempseed meal; RBP: Rice bran pellets; SBM: Soybean meal.

**Table 2:** Linear and multiple linear regression models using feedstuff fiber composition to predict in vitro true digestibility and fiber degradation.

Digestibility	Intercept	NDF	ADF	NDF × ADF	*p*-Value	RSE ^a^	Adjusted R^2^
IVTD	1052.30	−0.9325			<0.001	50.84	0.930
IVTD	1033.25		−1.5282		<0.001	70.22	0.866
IVTD	1003.32	−0.2904	−0.4220	−0.0010	<0.001	39.03	0.959
IVNDFD	810.31	−0.7528			<0.001	141.00	0.524
IVNDFD	855.15		−1.5183		<0.001	102.30	0.749
IVNDFD	987.97	−0.5981 ^b^	−2.2884	0.0024	<0.001	94.79	0.785
IVADFD	835.19	−0.7920			<0.001	131.00	0.586
IVADFD	881.91		−1.5952		<0.001	82.75	0.835
IVADFD	961.52	−0.2835 ^b^	−2.1878	0.0015	<0.001	77.90	0.854

Abbreviations: NDF: neutral detergent fiber (g/kg dry matter (DM)); ADF: acid detergent fiber (g/kg DM); IVTD: in vitro true digestibility (g/kg DM); IVNDFD: in vitro neutral detergent fiber digestibility (g/kg NDF); IVADFD: in vitro acid detergent fiber digestibility (g/kg ADF); ^a^: residual standard error; ^b^: variable not significant (*p* > 0.05).

**Table 3:** Estimation of in vitro true digestibility (IVTD) from feedstuff fiber composition using equine feces as incubation inoculum.

Model	*k*	LL	AICc	ΔAICc	AICcWt	Cum.Wt	Adjusted R^2^
IVTD~NDF × ADF	5	−231.74	474.97	0.00	1.0	1.0	0.959
IVTD~NDF	3	−244.97	496.51	21.54	0.0	1.0	0.930
IVTD~ADF	3	−259.82	526.22	51.25	0.0	1.0	0.866

Abbreviations: NDF: neutral detergent fiber (g/kg DM); ADF: acid detergent fiber (g/kg DM); IVTD: in vitro true digestibility (g/kg DM); *k*: (number of parameters) + 2; AICc: Akaike’s information criterion with correction factor; LL: log-likelihood; ΔAICc: Delta AICc from top-ranked model; AICcWt: individual AICc weight; Cum.Wt: cumulative weight of models.

**Table 4:** Estimation of in vitro neutral detergent fiber digestibility (IVNDFD) from feedstuff fiber composition using equine feces as incubation inoculum.

Model	*k*	LL	AICc	ΔAICc	AICcWt	Cum.Wt	Adjusted R^2^
IVNDFD~NDF × ADF	5	−272.56	556.61	0.00	0.89	0.89	0.785
IVNDFD~ADF	3	−277.12	560.82	4.21	0.11	1.00	0.749
IVNDFD~NDF	3	−291.89	590.35	33.73	0.00	1.00	0.524

Abbreviations: NDF: neutral detergent fiber (g/kg DM); ADF: acid detergent fiber (g/kg DM); IVNDFD: in vitro neutral detergent fiber digestibility (g/kg NDF); *k*: (number of parameters) + 2; AICc: Akaike’s information criterion with correction factor; LL: log-likelihood; ΔAICc: delta AICc from top-ranked model; AICcWt: individual AICc weight; Cum.Wt: cumulative weight of models.

**Table 5:** Estimation of in vitro acid detergent fiber digestibility (IVADFD) from feedstuff fiber composition using equine feces as incubation inoculum.

Model	*k*	LL	AICc	ΔAICc	AICcWt	Cum.Wt	Adjusted R^2^
IVADFD~NDF × ADF	5	−263.53	538.56	0.00	0.8	0.8	0.854
IVADFD~ADF	3	−267.38	541.33	2.77	0.2	1.0	0.834
IVADFD~NDF	3	−288.52	583.62	45.06	0.0	1.0	0.586

Abbreviations: NDF: neutral detergent fiber (g/kg dry matter (DM)); ADF: acid detergent fiber (g/kg DM); IVADFD: in vitro acid detergent fiber digestibility (g/kg ADF); *k*: (number of parameters) + 2; AICc: Akaike’s information criterion with correction factor; LL: log-likelihood; ΔAICc: delta AICc from top-ranked model; AICcWt: individual AICc weight; Cum.Wt: cumulative weight of models.

## Data Availability

The data presented in this study are available on request from the corresponding author. The data are not publicly available due to privacy of data as a companion study in which the data originated is currently under review.
